# Using Facebook to Recruit Young Australian Men Into a Cross-Sectional Human Papillomavirus Study

**DOI:** 10.2196/jmir.8739

**Published:** 2017-11-17

**Authors:** Roopa Das, Dorothy A Machalek, Edmund G Molesworth, Suzanne M Garland

**Affiliations:** ^1^ Melbourne Medical School University of Melbourne Parkville Australia; ^2^ Murdoch Children's Research Institute The Royal Children's Hospital Parkville Australia; ^3^ Department of Microbiology and Infectious Diseases The Royal Women's Hospital Melbourne Australia; ^4^ Department of Obstetrics and Gynaecology University of Melbourne Parkville Australia; ^5^ The Royal Children's Hospital Parkville Australia

**Keywords:** social media, Facebook, human papillomavirus, HPV, online recruitment, social networking, social networking sites, SNS

## Abstract

**Background:**

Young men can be difficult to engage in health research using traditional methods of recruitment. Social networking sites are increasingly being used to recruit participants into health research, due to their cost effectiveness, overall generalizability, and wide reach.

**Objective:**

The aim of this study was to determine the feasibility of using Facebook to recruit young Australian men into a human papillomavirus (HPV) prevalence study.

**Methods:**

We recruited male permanent residents of Australia, aged 18 to 35 years, into the HPV in Young Males (HYM) study through targeted advertising placed on Facebook. Consenting participants completed an online questionnaire and provided a self-collected penile swab for HPV DNA detection and genotyping. We compared sociodemographic characteristics of the study population with those of the general Australian male population, based on Australian 2011 census data.

**Results:**

Between February 2015 and February 2017, targeted Facebook advertisements reached 1,523,239 men, resulting in 41,811 clicks through to the study website, with 1072 (2.56%) converting to lodgment of an expression of interest. Of these, 681 (63.53%) provided written informed consent and 535 (78.6% of recruited participants) completed all the study requirements. Reasons for participating in the study included altruism, past history of HPV, gaining more knowledge about HPV or the vaccine, working in the health industry, and the monetary compensation. The average advertising cost per completed study participant was Aus $48. Compared with the census population, HYM study participants were more likely to be Australian born (*P*<.001), be from Victoria (*P*=.003) or the Australian Capital Territory (*P*=.004), reside in a major city (*P*<.001), and have completed undergraduate (*P*<.001) or postgraduate education (*P*<.001). HYM study participants were less likely to report being a current smoker (*P*=.03), but were more likely to identify as bisexual or homosexual (294/529, 55.6%, *P*<.001), than the general population.

**Conclusions:**

Using Facebook is a feasible and efficient strategy for the recruitment of men from across Australia for HPV testing. This method could be used for monitoring the impact of HPV vaccination. Additional targeting may achieve a sample that is broadly demographically representative of the Australian population. Future research should explore how the sexual risk behavior characteristics of populations recruited through Facebook compare with those of traditional recruitment methods.

## Introduction

Human papillomavirus (HPV) is one of the most common sexually transmitted infections globally. Infection with HPV is mostly asymptomatic; however, its clinical sequelae include genital warts, cervical cancer, and less commonly, a proportion of vaginal, vulvar, oral, oropharyngeal, anal, and penile cancers [1-3]. Many countries have introduced female HPV vaccination into their national immunization programs, with strong evidence showing reductions in vaccine-related-type HPV infections and disease where vaccine uptake has been high [4-6]. However, despite demonstrated trial efficacy [7,8] and the growing evidence that HPV also causes cancers in men (ie, penile, anal, and oropharyngeal), there is ongoing international debate on whether boys should be included in routine vaccination programs [9-14]. Consequently, only a handful of countries, including Australia, have implemented sex-neutral HPV vaccination approaches [15,16]. Monitoring the impact of HPV vaccination in men in these settings is thus important to document reductions in HPV-related biological end points (ie, genital HPV infections and cancers) caused by vaccine-targeted HPV genotypes and to inform whether routine vaccination of boys will result in any benefits above those that have already been achieved through existing female vaccination programs [17,18].

Measuring rates of genital HPV infection in men is challenging. HPV is very common, typically asymptomatic, and usually clears spontaneously. Furthermore, cancers associated with these infections are very rare. Thus, there are no population-based registries or screening programs, which could form the sampling frame for HPV vaccine monitoring studies in the general male population [19]. In view of this, to date, most direct estimates of HPV prevalence have been achieved through dedicated studies, whereby specimens for HPV testing are collected from opportunistic sampling of males recruited from clinical or community-based settings [17,20,21]. However, such studies are often expensive and require significant resources. Linking in with existing national surveillance activities, such as those implemented as part of the National Health and Nutrition Examination Surveys in the United States [22], is an ideal option for easily reaching populations of interest. However, in the absence of such frameworks, alternative and sustainable strategies are needed to collect genital specimens for the purpose of monitoring the impact of HPV vaccination on men in the long term.

Social networking sites (SNSs) present new opportunities for recruiting young men for health research, and they may have greater reach, be less time consuming, and be cheaper than traditional recruitment methods [23,24]. An estimated 99% of 18- to 29-year-old Australians use the social networking site Facebook, with 75% accessing this site at least once a day [25]. Facebook is also accessed throughout metropolitan and regional Australia, with at least 92% of people from each state of Australia using the platform [25]. This highlights the potential for access to a demographically and geographically diverse sample of participants. Advertising through SNSs has been shown to be successful for the recruitment of young females for surveys including health promotion and sexual health screening [24,26-31]. While SNSs have been effective in recruiting males into trials and sexual health promotion campaigns and surveys, both in Australia and internationally, including a recent US-based feasibility study aimed at recruiting males into a randomized controlled trial of HPV vaccination [32], little has been published with respect to study designs involving the collection of clinical specimens [33,34].

Our group has previously shown that Facebook was a cost-effective and fast method of recruiting young Australian women for home-based HPV testing [26]. In this study, we assessed the feasibility of using targeted Facebook advertising to recruit healthy Australian men aged 18 to 35 years into a HPV prevalence study.

## Methods

HPV in Young Males (HYM) is an ongoing study of HPV genotype prevalence in Australian men aged 18 to 35 years, recruited via Facebook. The study is part of a larger national surveillance program aiming to monitor circulating HPV genotypes in the Australian population, to evaluate the effectiveness of the Australian National HPV Vaccination Program [17]. The HYM study protocol was approved by the Royal Women’s Hospital Human Research and Ethics Committee (HREC Project number 14/22).

### Recruitment

**Figure 1:**
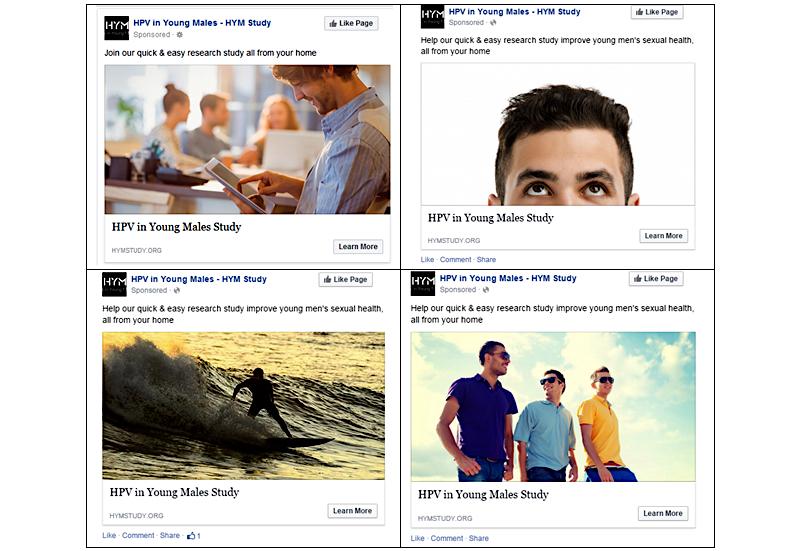
Examples of Facebook recruitment advertisements for the HPV in Young Males (HYM) study, as seen on News Feeds. HPV: human papillomavirus.

Recruitment to the study was based on strategies developed for the Vaccine Against Cervical Cancer Impact and Effectiveness (VACCINE) study, as previously described [6,35]. We recruited participants through targeted advertisements placed on Facebook (Facebook, Inc) between February 2015 and February 2017. Advertisements were targeted to men who were aged 18 to 35 years and living in Australia (we specifically selected the audience criteria on the Ads Manager of Facebook: users with an Australian IP address, male, and aged 18-35 years). Each advertisement contained a short text and image, examples of which are presented in Figure 1. Once clicking on the advertisement, participants were directed to the HYM password-protected website. Here potential participants could read about the study and register their expression of interest (EOI). Only men who registered their EOI were contacted by a study researcher. A telephone call established eligibility and obtained verbal consent. The inclusion criteria confirmed verbally were (1) male, (2) aged 18 to 35 years, (3) living permanently in Australia and eligible for a Medicare number, and (4) had been sexually active (ie, had ever had penetrative or oral sex).

After we obtained verbal consent, we sent a link to an online consent form to participants via SurveyMonkey (SurveyMonkey Inc). The consent consisted of 2 parts: consent for the study and consent to confirm the participant’s vaccination status with the Australian National HPV Vaccination Program Register. Once the online consent was completed, an online questionnaire and swab pack was sent out to participants. The online questionnaire, also done through SurveyMonkey, covered questions about basic demographics, sexual history, and knowledge and attitudes toward HPV and the HPV vaccines.

The swab pack contained everything needed to perform a self-collected penile swab in the privacy of a participant’s home. Participants were able to request and obtain their HPV DNA result. Once participants completed all the study requirements, they were compensated with an Aus $20 gift voucher for their time and effort. Participants were made aware of this compensation via the study website and during verbal consent. Among participants with outstanding study requirements, 3 reminders were sent via email, before the person was deemed lost from the study.

### Statistical Analysis

We used Facebook metrics to calculate the amount spent on the advertising campaign. Facebook used a bidding system to display advertisements, with a cost allocated each time an advertisement was clicked [24]. Demographics for analyses were as follows: age (grouped as 18-24 years, 25-29 years, and 30-35 years), Australian state or territory of residence, gender identity, education, geographical remoteness (major city, inner regional, or outer regional or remote [36]), Aboriginal or Torres Strait Island status, country of birth, relationship status (single or casual, or committed relationship, which included marriage and de facto relationships), smoking, income, and employment status. We compared demographics with the 2011 census data on men aged 18 to 35 years, from the Australian Bureau of Statistics [37], and the Second Australian Study of Health and Relationships published by the Commonwealth Scientific and Industrial Research Organisation [38]. Chi-square, 95% CIs, and *P* values <.05 were used to assess significance. Stata 14 (StataCorp LLC) was used to perform statistical analyses.

## Results

Between February 2015 and February 2017, we simultaneously ran one campaign containing 5 advertisements, which reached 1,523,239 Australian male Facebook users within the target age range. In that time, the campaign was presented to users 7,029,581 times. The advertisements were seen on mobile phones or tablets (n=687,682, 45.15%), desktop computers (n=336,813, 22.11%), and other devices (n=498,744, 32.74%). There were 41,811 clicks (0.59% of total advertisement presentations) on the advertisements, resulting in 1072 (2.56% of clicks) EOIs. The advertisements were shown at all times and were viewed on average 4.61 times per day (the number varied depending on the amount of money placed on the advertisement).

Of the 1072 participants who submitted an EOI, 681 (63.53% of EOIs) provided verbal and written consent and were recruited into the study. The primary reasons for expressing an interest to participate in the study were altruism (n=577, 53.82%); being interested in the area (ie, sexual health, HPV or vaccination) (n=180, 16.79%); working in medical research or a health-related area (n=129, 12.03%); wanting to know their HPV result (n=64, 5.97%); and having a history of, or knowing someone with a history of, HPV infection or related disease (eg, genital warts or cervical lesions) (n=96, 8.96%). Only 2.42% (n=26) of men reported the monetary compensation as a reason for wanting to participate.

Of the remaining 391 (36.47%) EOIs, the majority (n=352, 90.0%) could not be contacted to obtain verbal or written consent and were subsequently lost from the study. A further 37 (9.5%) were deemed ineligible after telephone contact, and 2 (0.5%) were yet to be contacted. The total amount of money spent on the advertisement campaign in this time period was Aus $25,687.64, with the average cost per click being Aus $0.61. The cost per EOI translated to Aus $23.96. The cost per completed participant was Aus $48.01.

Table 1 presents the demographic characteristics of the 1072 participants who submitted an EOI. The median age of respondents was 26 years (interquartile range 23-30 years). The vast majority of EOIs (n=929, 86.66%) were generated directly from clicking on Facebook advertisements. Of the remaining 143 EOIs, 84 (7.8% of the total) were derived from referrals by friends or family, 20 (1.9%) from media (eg, Flipboard), and 39 (3.6%) from other sources. The vast majority of men who submitted an EOI resided in the eastern states of Australia (882/1059, 83.29%). There were no significant differences in age, how participants heard of the study, and the state in which they resided between respondents who were recruited into the study and those who were not.

Of the 681 recruited participants, 535 (78.6%) had completed the study at the time of writing this report. The median time from the date of EOI to the date that the swab pack was returned was 5 weeks (interquartile range 3-8). Of the remaining 146 participants, 91 (13.3%) were in the process of completing the study, and 55 (8.1%) did not return the swab pack after multiple reminders and were therefore deemed lost to follow-up. There were no differences in age between participants who completed the study and those who did not return their swab pack.

Table 2 presents the sociodemographic characteristics of the 535 HYM study participants who completed the study compared with those of the general Australian age-matched male population. Compared with the general population, there was a significantly higher proportion of men aged 25 to 29 years of age participating in the study (*P*=.003). There was also a significant difference in location of residence between men who completed the study and the general population. Men residing in Victoria (*P*=.003) and the Australian Capital Territory (*P*=.004) were overrepresented in our cohort, while those living in Western Australia (*P*<.001) were underrepresented.

**Table 1:** Demographic characteristics of 1072 men who expressed interest to participate in the HPV^a^ in Young Males (HYM) study.

Characteristics		Overall (N=1072)^b^		Recruited into HYM^c^		Not recruited into HYM (n=391)		*P* value^d^
		n	%	n	%	n	%	
**Age group (years)**								.07
	18-24	384	36.23	240	35.2	144	38.0	
	25-29	376	35.47	232	34.1	144	38.0	
	30-35	300	28.30	209	30.7	91	24.0	
**Heard about the study from...**								.09
	Facebook ad	929	86.66	590	86.6	339	86.7	
	Friend or family	84	7.84	59	8.7	25	6.4	
	Media	20	1.86	8	1.2	12	3.1	
	Other^e^	39	3.64	24	3.5	15	3.8	
**Australian state of residence**								.56
	Victoria	318	30.03	201	29.5	117	31.0	
	New South Wales	311	29.37	203	29.8	108	28.6	
	Queensland	216	20.40	143	21.0	73	19.3	
	Western Australia	85	8.03	47	6.9	38	10.0	
	South Australia	59	5.57	37	5.4	22	5.8	
	Australian Capital Territory	37	3.49	25	3.7	12	3.2	
	Tasmania	22	2.08	17	2.5	5	1.3	
	Northern Territory	11	1.03	8	1.2	3	0.8	

^a^HPV: human papillomavirus.

^b^Numbers do not always add up to 1072 due to small amount of missing data.

^c^Participants who provided verbal and written consent.

^d^Chi-square test was used to determine *P* value.

^e^Other includes hearing about the study from university, work, or health care settings.

**Table 2:** Demographic characteristics of 535 HPV^a^ in Young Males (HYM) study participants compared with the general Australian male population aged 18-35 years.

Characteristics		Completed the study (n=535)^b^			General population (%)^c^	*P* value^d^
		n	%	95% CI		
**Age group (years)**						
	18-24 years	193	36.1	32.0-40.3	38.9	.18
	25-29 years	184	34.4	30.4-38.6	28.5	.003
	30-35 years	158	29.5	25.7-33.6	32.6	.13
**Australian state of residence**						
	Victoria	167	31.2	27.3-35.3	25.5	.003
	New South Wales	161	30.1	26.2-34.2	31.3	.55
	Queensland	109	20.4	17.0-24.0	19.9	.77
	Western Australia	33	6.2	4.3-8.6	11.1	<.001
	South Australia	24	4.5	2.9-6.6	7.1	.02
	Australian Capital Territory	20	3.7	2.3-5.7	2.0	.004
	Tasmania	14	2.6	1.4-4.4	1.9	.24
	Northern Territory	7	1.3	0.5-2.7	1.2	.86
**Education**						
	Year 12 or below	110	20.6	17.2-24.2	45.9	<.001
	Undergraduate or certificate	340	63.6	59.3-67.6	50.1	<.001
	Postgraduate	79	14.8	11.9-18.1	4.0	<.001
**Geographic region**						
	Major cities	441	82.4	78.9-85.6	75.1	<.001
	Inner regional	54	10.1	7.7-13.0	14.8	.002
	Outer regional or remote	40	7.5	5.4-10.0	10.1	.045
**Aboriginal or Torres Strait Islander**						
	Yes	15	2.8	1.6-4.6	2.9	.92
	No	514	97.2	95.3-98.4	97.1	.92
**Country of birth**						
	Australia	439	83.0	79.5-86.1	72.8	<.001
	Other	90	17.0	13.9-20.5	27.2	<.001
**Employment status**						
	Full-time	306	57.2	53.7-62.3	61.7	.09
	Part-time	141	26.4	23.0-30.8	16.6	<.001
	Not in the labor force^e^	47	8.8	6.6-11.7	14.9	<.001
	Unemployed	33	6.2	4.3-8.7	6.8	.62
**Gender identity**						
	Heterosexual	235	44.4	40.1-48.8	96.7	<.001
	Bisexual or homosexual	294	55.6	51.2-59.9	3.3	<.001
**Relationship status**						
	Single or casual	256	49.2	44.5-53.2	60.6	<.001
	Committed	272	50.8	471-55.9	39.4	<.001
**Regular cigarette smoker**						
	Never	353	66.7	62.5-70.7	53.7	<.001
	Past	94	17.8	14.6-21.3	26.4	<.001
	Current	82	15.5	12.5-18.9	19.9	.03

^a^HPV: human papillomavirus.

^b^Numbers do not always add up to 535 due to small amount of missing data.

^c^Target population data sourced from the 2011 census by the Australian Bureau of Statistics and Second Australian Study of Health and Relationships [36,37].

^d^Chi-square test was used to determine *P* values.

^e^Not in the labor force and not looking for work included full-time caregivers or students.

A larger proportion of HYM study participants had higher education (*P*<.001), lived in major cities in Australia (*P*<.001), were born in Australia (*P*<.001), had never smoked cigarettes or had quit smoking (*P*<.001 never and past; *P*=.03 current), were in a committed relationship (*P*<.001), or worked part-time (*P*<0.001). Study participants were also more likely to identify as gay or bisexual than the underlying population (*P*<.001). There was no significant difference in indigenous status between the 2 populations (*P*=.92).

## Discussion

### Principal Findings

In this study, we used targeted Facebook advertising to recruit Australian men aged 18 to 35 years into a cross-sectional HPV genotype prevalence study. Consenting participants were required to complete an online questionnaire and return a self-collected penile swab for HPV detection and genotyping. Over a 2-year study period, 64% of the 1072 men who submitted an EOI were recruited, and of these, the majority (79%) completed the study requirements, making this an acceptable and effective strategy for collecting specimens, which are not routinely collected clinically. However, while the strategy was convenient and had the potential to reach a large cross-section of the population, the sociodemographic characteristics of men who participated in the HYM study were proportionally different from those of the general population of the same age as measured by the census, suggesting that additional targeting is needed to achieve a sample that is broadly demographically representative of the Australian population.

Online recruitment methods have been used for behavioral surveys among men, but few studies have reported their use for subsequent self-collection of a specimen [33,34,39]. The HYM study findings suggest that Facebook can be used to recruit young men for HPV testing and that social media could provide a potential alternative avenue for targeting young men for sexually transmitted infection screening in general. A recent Australian study showed that home-based sexually transmitted infection screening was popular among young men due to such screening being free and convenient [39]. Our study had a high return rate of self-collected specimens (79%), highlighting the feasibility of sending swabs for self-collection by mail. The high return rates are in contrast to those reported in some published studies using home-based collection kits for sexually transmitted infection screening (48.2% in Australia, 40% in Sweden, 7.8% in the United States, and 33% in the Netherlands) [39-42]. This may reflect differences in study design, namely, the option of returning specimens by mail, rather than directly to a collection center, in addition to providing multiple reminders to return the pack, rather than none or only 1 reminder.

While using Facebook was an efficient strategy for targeting men for recruitment, the cost per participant recruited was Aus $24 higher than with women participating in a similar HPV vaccine effectiveness study [26]. Our study reached more men than the number of women reached by the VACCINE study (1,523,239 men and 984,159 women), despite the shorter duration of our campaign, and had a higher click-through rate to the study website (0.6% compared with 0.04% for the VACCINE study’s female participants). This likely reflected the broader eligibility criteria of this study, with all Australian men aged 18 to 35 years targeted for recruitment, compared with the VACCINE study, where recruitment was limited to 18- to 25-year-old women living in Victoria [26]. Despite this, once on the study website, a lower proportion of men submitted an EOI compared with the female study (3% versus 6% respectively). We did not collect reasons for not wanting to participate in the HYM study; however, we hypothesize that the potentially confronting study requirements may have, to some extent, discouraged some men from taking part. Self-collecting a sample of cells from the surface of the penis is an acceptable and common form of specimen collection for HPV testing in epidemiological studies [20,43-45]. However, the method is not routine clinical practice and is likely to be unfamiliar to most men [46].

While to the best of our knowledge HYM is the first study to recruit young Australian men into an HPV prevalence survey through advertising on SNSs, elements of the study findings, with respect to cohort characteristics, are consistent with previous published research. For example, we found that our cohort was more educated than the general population. Studies recruiting through SNSs commonly recruit cohorts that are more highly educated than the general population [24,26,28,47]. Likewise, our cohort consisted of a larger proportion of younger men, which is consistent with the population of users of SNSs, who tend to be younger than the general population [25]. However, more men from Victoria and the Australian Capital Territory, and fewer men from Western Australia, submitted an EOI and completed the study. Victorian men may have been more inclined to participate, as this study was based in Victoria. Further, logistical reasons such as the time difference between the eastern states and Western Australia posed a large difficulty in receiving verbal consent, contributing to lower numbers of participants from Western Australia.

Similarly to previous research, we found that a significantly higher proportion of men who identified as homosexual or bisexual participated in the study [32], despite the broad eligibility criteria and attempts to achieve sexual preference neutrality in both the images and text displayed on the recruitment advertisements (see Figure 1). The use of Internet-based social media has been widely promoted as a feasible strategy for the recruitment of men who have sex with men. Their overrepresentation observed in the HYM study may have been due to heightened knowledge and concern regarding HPV among this population, who are at increased risk of HPV-related disease [42,43,48]. This highlights the need for additional targeted advertising to achieve a sample that is broadly demographically representative of the general population. A benefit of using Facebook is the ability to readily modify how one targets specific cohorts for recruitment by adjusting the wording, images, and placement of the advertising, as has been shown in previous studies [26,49].

The main question arising from the study, with respect to the suitability of using SNSs such as Facebook for long-term HPV monitoring, is the potential impact of this form of convenience sampling on the stability of the population under surveillance, as well as the external validity of any observed HPV prevalence estimates. Future research should further explore how the demographic and sexual risk behavior characteristics of populations recruited through Facebook compare with traditional recruitment methods (ie, clinical or community-based samples), and monitor how samples of young men recruited via Facebook change over time with respect to demographic and sexual behavior characteristics.

Furthermore, it is important to note that, while Facebook is the most popular SNS in Australia, the use of other SNSs such as Twitter, Instagram, and Snapchat is on the rise [25]. Of note, SNSs that use imaging and visual components such as Snapchat and Instagram are particularly popular among younger Australians aged less than 30 years. Therefore, it will be interesting in the future for studies to assess the effectiveness of recruiting younger participants through other SNSs.

### Conclusions

This study demonstrated that using Facebook was an effective strategy in recruiting young Australian men for home-based HPV testing, with high return rates of specimens. This modality could potentially be used to monitor the prevalence of circulating HPV genotypes in Australia to help determine the impact of routine HPV vaccination. Moreover, home-based swab collection is a potential avenue for future health screening among men.

Our study population was different from the general population. However, through targeting of Facebook advertisements, and collecting demographic and sexual behavior data, we can hope to mitigate this issue in future analyses. Further research is needed in assessing the demographic characteristics of men recruited through Facebook over time and compared with men recruited through more traditional methods to assess the viability of using Facebook in long-term HPV monitoring. With the exponentially fast rate of change among SNS uptake, more research is needed to inform the feasibility of using other SNSs to recruit men into health studies.

## Abbreviations

EOI: expression of interest
HPV: human papillomavirus
HYM: HPV in Young Males
SNS: social networking site
VACCINE: Vaccine Against Cervical Cancer Impact and Effectiveness

## References

[1] Koutsky L (1997). Epidemiology of genital human papillomavirus infection. Am J Med.

[2] Georgousakis M, Jayasinghe S, Brotherton J, Gilroy N, Chiu C, Macartney K (2012). Population-wide vaccination against human papillomavirus in adolescent boys: Australia as a case study. Lancet Infect Dis.

[3] Centers for Disease Control an Prevention (1999). Prevention of genital HPV infection and sequelae: report of an external consultants' meeting.

[4] Tabrizi SN, Brotherton JML, Kaldor JM, Skinner SR, Liu B, Bateson D, McNamee K, Garefalakis M, Phillips S, Cummins E, Malloy M, Garland SM (2014). Assessment of herd immunity and cross-protection after a human papillomavirus vaccination programme in Australia: a repeat cross-sectional study. Lancet Infect Dis.

[5] Tabrizi SN, Brotherton JML, Kaldor JM, Skinner SR, Cummins E, Liu B, Bateson D, McNamee K, Garefalakis M, Garland SM (2012). Fall in human papillomavirus prevalence following a national vaccination program. J Infect Dis.

[6] Young EJ, Tabrizi SN, Brotherton JM, Wark JD, Pyman J, Saville M, Wrede CD, Jayasinghe Y, Tan J, Gertig DM, Pitts M, Garland SM (2013). Measuring effectiveness of the cervical cancer vaccine in an Australian setting (the VACCINE study). BMC Cancer.

[7] Hillman RJ, Giuliano AR, Palefsky JM, Goldstone S, Moreira ED, Vardas E, Aranda C, Jessen H, Ferris DG, Coutlee F, Marshall JB, Vuocolo S, Haupt RM, Guris D, Garner EIO (2012). Immunogenicity of the quadrivalent human papillomavirus (type 6/11/16/18) vaccine in males 16 to 26 years old. Clin Vaccine Immunol.

[8] Goldstone SE, Jessen H, Palefsky JM, Giuliano AR, Moreira ED, Vardas E, Aranda C, Hillman RJ, Ferris DG, Coutlee F, Marshall JB, Vuocolo S, Haupt RM, Guris D, Garner E (2013). Quadrivalent HPV vaccine efficacy against disease related to vaccine and non-vaccine HPV types in males. Vaccine.

[9] Elbasha EH, Dasbach EJ (2010). Impact of vaccinating boys and men against HPV in the United States. Vaccine.

[10] Marty R, Roze S, Bresse X, Largeron N, Smith-Palmer J (2013). Estimating the clinical benefits of vaccinating boys and girls against HPV-related diseases in Europe. BMC Cancer.

[11] Kulasingam S, Connelly L, Conway E, Hocking JS, Myers E, Regan DG, Roder D, Ross J, Wain G (2007). A cost-effectiveness analysis of adding a human papillomavirus vaccine to the Australian National Cervical Cancer Screening Program. Sex Health.

[12] Kim JJ, Andres-Beck B, Goldie SJ (2007). The value of including boys in an HPV vaccination programme: a cost-effectiveness analysis in a low-resource setting. Br J Cancer.

[13] Burger EA, Sy S, Nygård M, Kristiansen IS, Kim JJ (2014). Prevention of HPV-related cancers in Norway: cost-effectiveness of expanding the HPV vaccination program to include pre-adolescent boys. PLoS One.

[14] Pearson AL, Kvizhinadze G, Wilson N, Smith M, Canfell K, Blakely T (2014). Is expanding HPV vaccination programs to include school-aged boys likely to be value-for-money: a cost-utility analysis in a country with an existing school-girl program. BMC Infect Dis.

[15] Stanley M (2014). HPV vaccination in boys and men. Hum Vaccin Immunother.

[16] Bruni L, Barrionuevo-Rosa L, Albero G, Serrano B, Mena M, Gomez D, Munoz J, Bosch F, de Sanjose S (2017). Human papillomavirus and related disease report in Austria. Summary report 2017.

[17] Machalek DA, Chow EPF, Garland SM, Wigan R, Cornall AM, Fairley CK, Kaldor JM, Hocking JS, Williams H, McNulty A, Bell C, Marshall L, Ooi C, Chen MY, Tabrizi SN (2017). Human papillomavirus prevalence in unvaccinated heterosexual men after a national female vaccination program. J Infect Dis.

[18] Garland SM, Molesworth EG, Machalek DA, Cornall AM, Tabrizi SN (2015). How to best measure the effectiveness of male human papillomavirus vaccine programmes?. Clin Microbiol Infect.

[19] Kavanagh K, Sinka K, Cuschieri K, Love J, Potts A, Pollock KGJ, Cubie H, Donaghy M, Robertson C (2013). Estimation of HPV prevalence in young women in Scotland; monitoring of future vaccine impact. BMC Infect Dis.

[20] Giuliano AR, Lee J, Fulp W, Villa LL, Lazcano E, Papenfuss MR, Abrahamsen M, Salmeron J, Anic GM, Rollison DE, Smith D (2011). Incidence and clearance of genital human papillomavirus infection in men (HIM): a cohort study. Lancet.

[21] Nyitray AG, Carvalho DSRJ, Baggio ML, Lu B, Smith D, Abrahamsen M, Papenfuss M, Villa LL, Lazcano-Ponce E, Giuliano AR (2011). Age-specific prevalence of and risk factors for anal human papillomavirus (HPV) among men who have sex with women and men who have sex with men: the HPV in men (HIM) study. J Infect Dis.

[22] Gargano JW, Unger ER, Liu G, Steinau M, Meites E, Dunne E, Markowitz LE (2017). Prevalence of genital human papillomavirus in males, United States, 2013-2014. J Infect Dis.

[23] Prescott TL, Phillips IG, DuBois LZ, Bull SS, Mustanski B, Ybarra ML (2016). Reaching adolescent gay, bisexual, and queer men online: development and refinement of a national recruitment strategy. J Med Internet Res.

[24] Fenner Y, Garland SM, Moore EE, Jayasinghe Y, Fletcher A, Tabrizi SN, Gunasekaran B, Wark JD (2012). Web-based recruiting for health research using a social networking site: an exploratory study. J Med Internet Res.

[25] Sensis (2016). Sensis social media report 2016: how Australian people and businesses are using social media.

[26] Subasinghe AK, Nguyen M, Wark JD, Tabrizi SN, Garland SM (2016). Targeted Facebook advertising is a novel and effective method of recruiting participants into a human papillomavirus vaccine effectiveness study. JMIR Res Protoc.

[27] Kapp JM, Peters C, Oliver DP (2013). Research recruitment using Facebook advertising: big potential, big challenges. J Cancer Educ.

[28] Mishra GD, Hockey R, Powers J, Loxton D, Tooth L, Rowlands I, Byles J, Dobson A (2014). Recruitment via the Internet and social networking sites: the 1989-1995 cohort of the Australian Longitudinal Study on Women's Health. J Med Internet Res.

[29] Ahmed N, Jayasinghe Y, Wark JD, Fenner Y, Moore EE, Tabrizi SN, Fletcher A, Garland SM (2013). Attitudes to Chlamydia screening elicited using the social networking site Facebook for subject recruitment. Sex Health.

[30] Gunasekaran B, Jayasinghe Y, Fenner Y, Moore EE, Wark JD, Fletcher A, Tabrizi SN, Garland SM (2013). Knowledge of human papillomavirus and cervical cancer among young women recruited using a social networking site. Sex Transm Infect.

[31] Miyagi E, Motoki Y, Asai-Sato M, Taguri M, Morita S, Hirahara F, Wark JD, Garland SM (2014). Web-based recruiting for a survey on knowledge and awareness of cervical cancer prevention among young women living in Kanagawa prefecture, Japan. Int J Gynecol Cancer.

[32] Raviotta JM, Nowalk MP, Lin CJ, Huang H, Zimmerman RK (2014). Using Facebook™ to recruit college-age men for a human papillomavirus vaccine trial. Am J Mens Health.

[33] Pedrana A, Hellard M, Gold J, Ata N, Chang S, Howard S, Asselin J, Ilic O, Batrouney C, Stoove M (2013). Queer as F**k: reaching and engaging gay men in sexual health promotion through social networking sites. J Med Internet Res.

[34] Nguyen P, Gold J, Pedrana A, Chang S, Howard S, Ilic O, Hellard M, Stoove M (2013). Sexual health promotion on social networking sites: a process evaluation of The FaceSpace Project. J Adolesc Health.

[35] Osborne SL, Tabrizi SN, Brotherton JML, Cornall AM, Wark JD, Wrede CD, Jayasinghe Y, Gertig DM, Pitts MK, Garland SM (2015). Assessing genital human papillomavirus genoprevalence in young Australian women following the introduction of a national vaccination program. Vaccine.

[36] Commonwealth Department of Health and Aged Care Measuring Remoteness: Accessibility/Remoteness Index of Australia (ARIA) - revised edition 2001.

[37] Australian Bureau of Statistics (2013). 2011 census data.

[38] Richters J, Altman D, Badcock PB, Smith AMA, de Visser RO, Grulich AE, Rissel C, Simpson JM (2014). Sexual identity, sexual attraction and sexual experience: the Second Australian Study of Health and Relationships. Sex Health.

[39] Martin L, Freedman E, Burton L, Rutter S, Knight V, D'Amato A, Murray C, Drysdale J, Harvey S, McNulty A (2009). The C-project: use of self-collection kits to screen for Chlamydia trachomatis in young people in a community-based health promotion project. Sex Health.

[40] Novak DP, Karlsson RB (2006). Simplifying chlamydia testing: an innovative Chlamydia trachomatis testing approach using the internet and a home sampling strategy: population based study. Sex Transm Infect.

[41] Scholes D, Heidrich FE, Yarbro P, Lindenbaum JE, Marrazzo JM (2007). Population-based outreach for Chlamydia screening in men: results from a randomized trial. Sex Transm Dis.

[42] van Bergen J, Götz HM, Richardus JH, Hoebe CJPA, Broer J, Coenen AJT, PILOT CT study group (2005). Prevalence of urogenital Chlamydia trachomatis increases significantly with level of urbanisation and suggests targeted screening approaches: results from the first national population based study in the Netherlands. Sex Transm Infect.

[43] Giuliano AR, Palefsky JM, Goldstone S, Moreira ED, Penny ME, Aranda C, Vardas E, Moi H, Jessen H, Hillman R, Chang Y, Ferris D, Rouleau D, Bryan J, Marshall JB, Vuocolo S, Barr E, Radley D, Haupt RM, Guris D (2011). Efficacy of quadrivalent HPV vaccine against HPV Infection and disease in males. N Engl J Med.

[44] Goldstone S, Palefsky JM, Giuliano AR, Moreira ED, Aranda C, Jessen H, Hillman RJ, Ferris DG, Coutlee F, Liaw K, Marshall JB, Zhang X, Vuocolo S, Barr E, Haupt RM, Guris D, Garner EIO (2011). Prevalence of and risk factors for human papillomavirus (HPV) infection among HIV-seronegative men who have sex with men. J Infect Dis.

[45] Zou H, Tabrizi SN, Grulich AE, Hocking JS, Bradshaw CS, Cornall AM, Morrow A, Prestage G, Law MG, Garland SM, Chen MY, Fairley CK (2015). Site-specific human papillomavirus infection in adolescent men who have sex with men (HYPER): an observational cohort study. Lancet Infect Dis.

[46] Royal Australian College of General Practitioners Guidelines for preventative activities in general practice 2016.

[47] Harris ML, Loxton D, Wigginton B, Lucke JC (2015). Recruiting online: lessons from a longitudinal survey of contraception and pregnancy intentions of young Australian women. Am J Epidemiol.

[48] Vardas E, Giuliano AR, Goldstone S, Palefsky JM, Moreira ED, Penny ME, Aranda C, Jessen H, Moi H, Ferris DG, Liaw K, Marshall JB, Vuocolo S, Barr E, Haupt RM, Garner EIO, Guris D (2011). External genital human papillomavirus prevalence and associated factors among heterosexual men on 5 continents. J Infect Dis.

[49] Reiter PL, Katz ML, Bauermeister JA, Shoben AB, Paskett ED, McRee A (2017). Recruiting young gay and bisexual men for a human papillomavirus vaccination intervention through social media: the effects of advertisement content. JMIR Public Health Surveill.

